# Supplementary figures and images for: Colonoscopy polyp classification via enhanced scattering wavelet Convolutional Neural Network
Source: PLoS One. 2024 Oct 11;19(10):e0302800. doi: 10.1371/journal.pone.0302800 (PMC11469526; doi:10.1371/journal.pone.0302800)

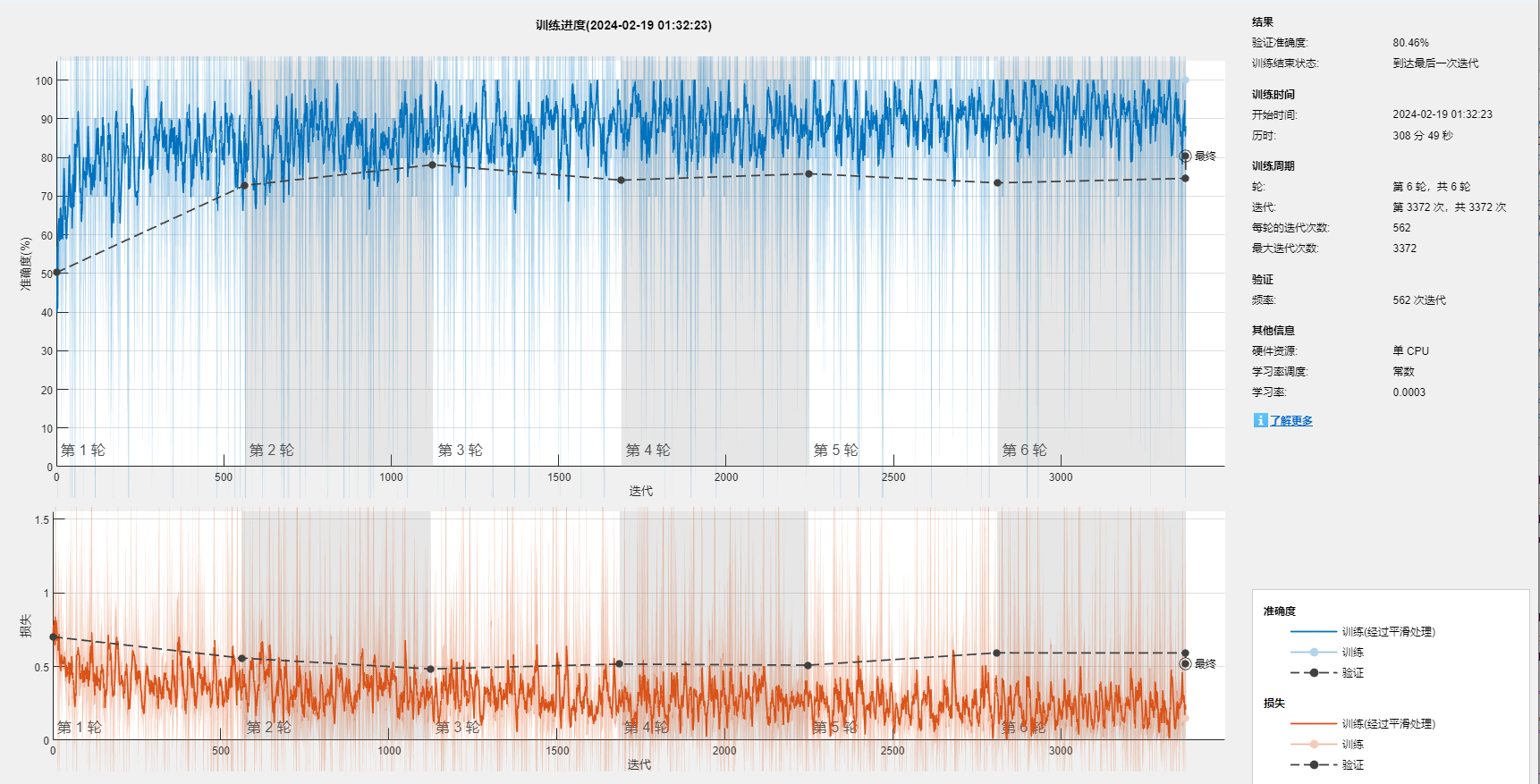

Supplement: S1 File — (ZIP) [file pone.0302800.s001.zip › experiment Result/DenseNet201.png]

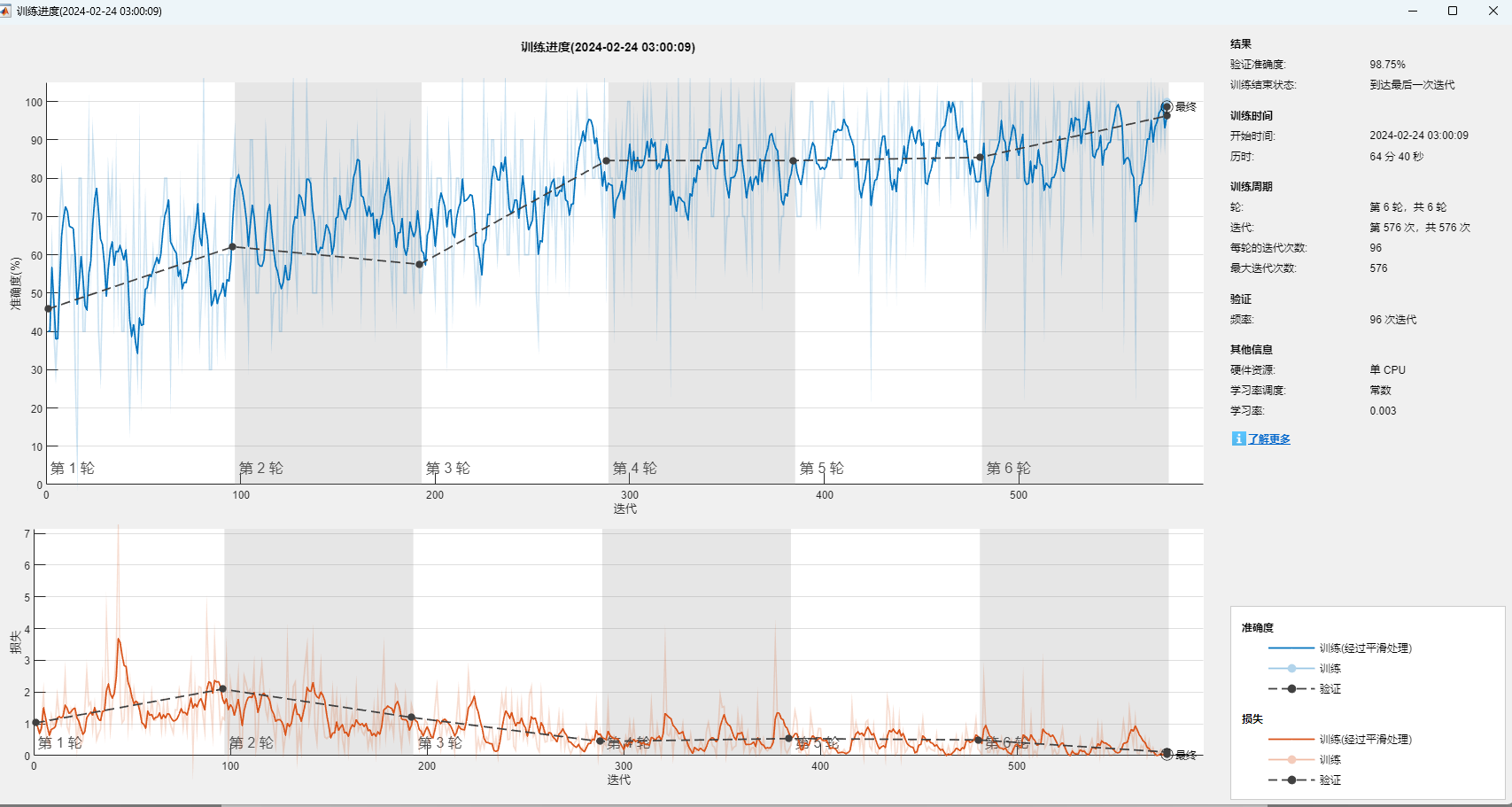

Supplement: S1 File — (ZIP) [file pone.0302800.s001.zip › experiment Result/DenseNet2010225.png]

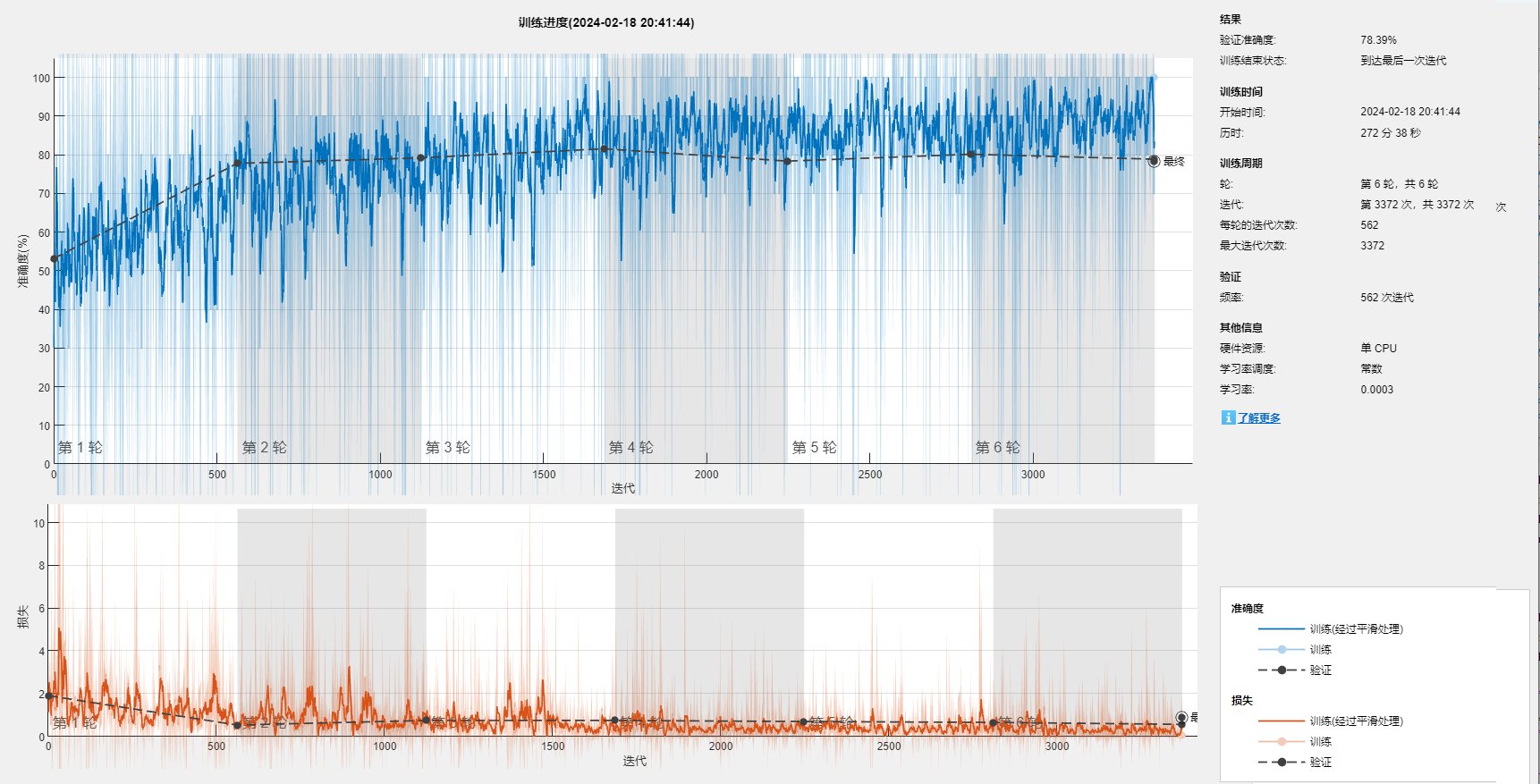

Supplement: S1 File — (ZIP) [file pone.0302800.s001.zip › experiment Result/ResNet101.png]

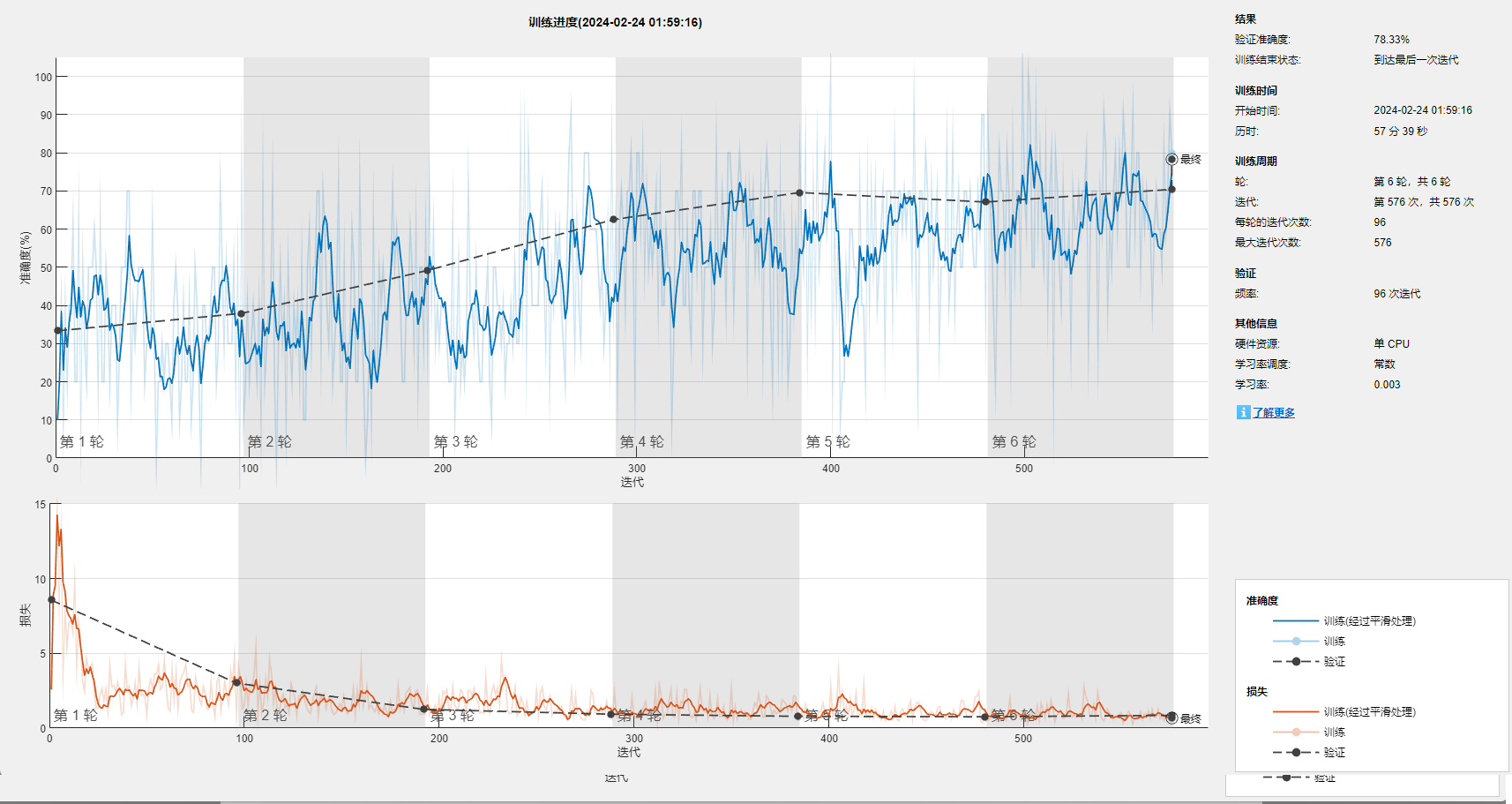

Supplement: S1 File — (ZIP) [file pone.0302800.s001.zip › experiment Result/ResNet1010224.png]

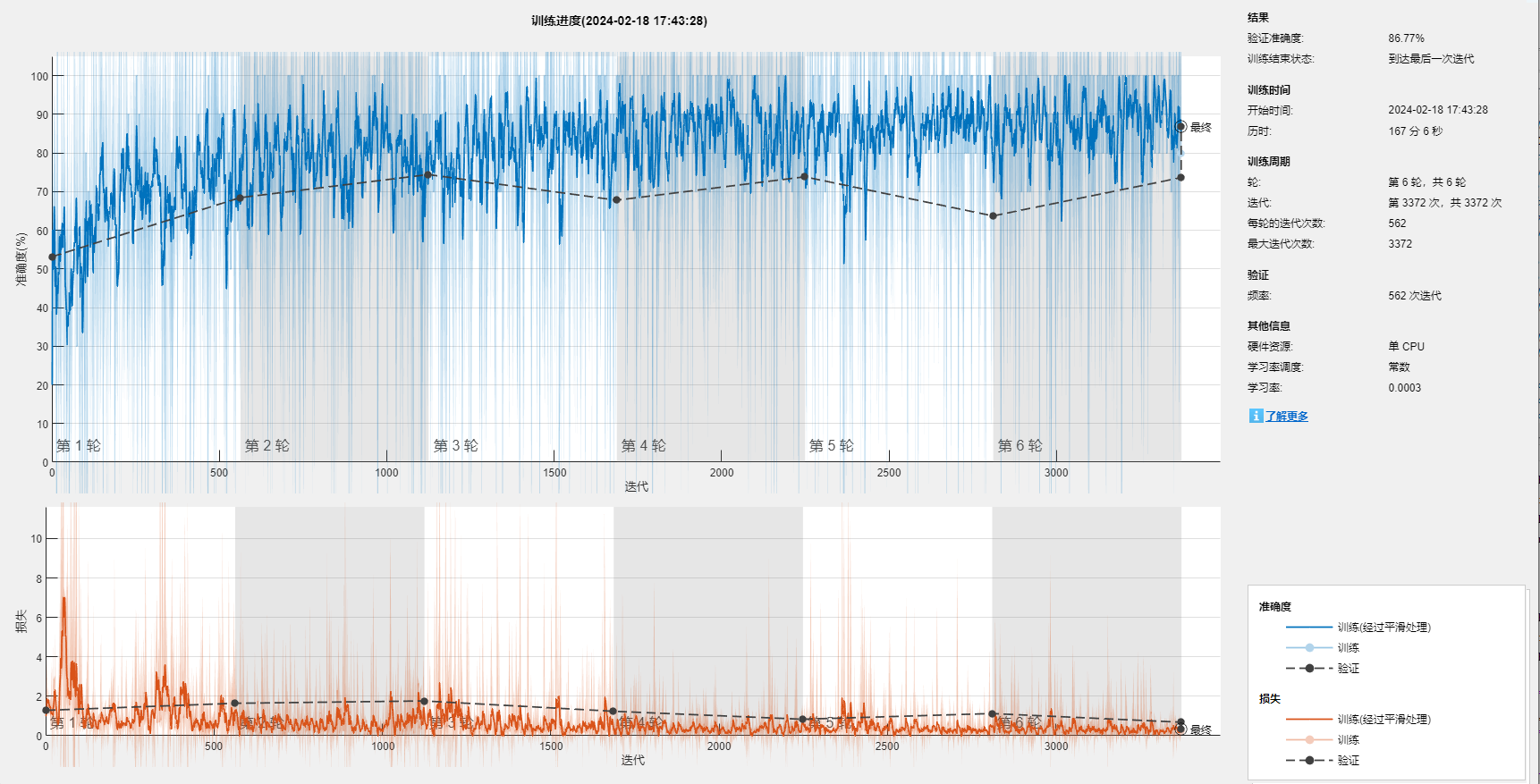

Supplement: S1 File — (ZIP) [file pone.0302800.s001.zip › experiment Result/ResNet50.png]

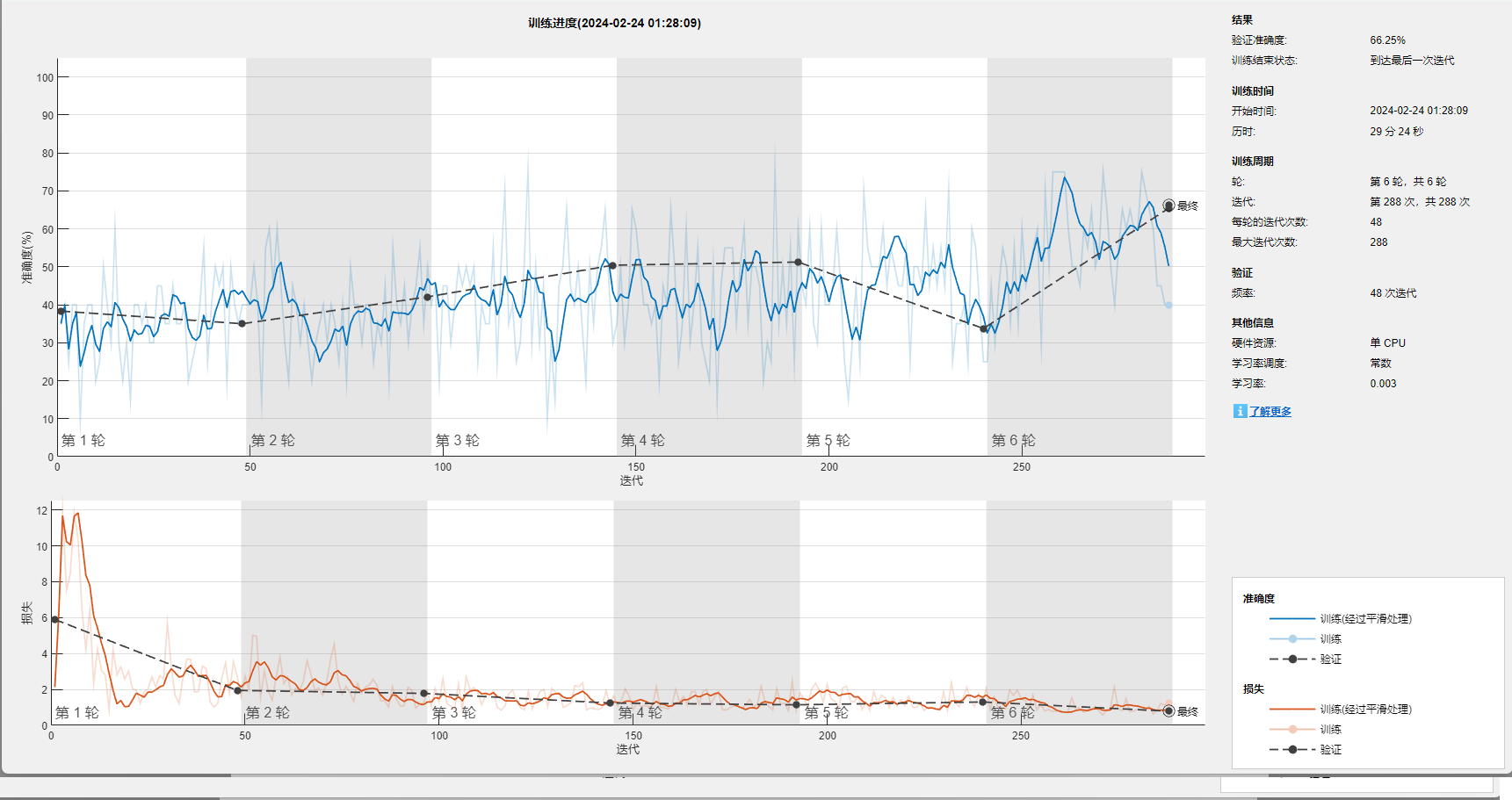

Supplement: S1 File — (ZIP) [file pone.0302800.s001.zip › experiment Result/ResNet500224.png]
